# Global Governing Bodies: A Pathway for Gene Drive Governance for Vector Mosquito Control

**DOI:** 10.4269/ajtmh.19-0941

**Published:** 2020-08-03

**Authors:** Adam Kelsey, Drusilla Stillinger, Thai Binh Pham, Jazmin Murphy, Sean Firth, Rebeca Carballar-Lejarazú

**Affiliations:** Department of Microbiology and Molecular Genetics, University of California, Irvine, California

## Abstract

Gene drive technologies represent powerful tools to develop vector control strategies that will complement the current approaches to mitigate arthropod-borne infectious diseases. The characteristics of gene drive technologies have raised additional concerns to those for standard genetically engineered organisms. This generates a need for adaptive governance that has not been met yet because of the rapid rate of progress in gene drive research. For the eventual release of gene drive insects into wild populations, an international governance network would be helpful in guiding scientists, stakeholders, public opinion, and affected communities in its use. We examined the current institutions and governing bodies among various continents that could have an impact on gene drive governance or the potential to adapt to its future use. Possible governance strategies also are proposed that seek to bridge gaps and promote an ethically sound policy framework. Ideally, governance strategies should be developed before or at the same pace as gene drive research to anticipate field releases and maximize their impact as a public health tool. However, this is not likely to happen as it takes years to develop global accords, and some countries may choose to move ahead independently on the new technology.

## INTRODUCTION

Mosquito-borne diseases impose a massive burden on human health. The existing vector control strategies are insufficient to support sustainable activities toward disease reduction. New genetic technologies, such as CRISPR-Cas9 systems, can potentially contribute to eliminate some of the most devastating infectious diseases including malaria, dengue, and other arboviruses by targeting the vector populations.^1^ Cas9-mediated gene-editing is an efficient and adaptable platform for gene drive strategies and together with other gene drive mechanisms in “active genetics” has the potential to quickly alter the genetics of large populations of species with high reproductive rates.^2^ However, gene drives have to establish a precedent of efficacy in the field while mitigating concerns about long-term stability, reversibility, and spatial confinement. These combined challenges are similar to those seen in standard genetically modified organisms (GMOs), but the enhanced aspects of population spread by drive organisms may require adaptive regulatory policy and communication between governing bodies to ensure proper field testing and implementation for disease control. In addition, the rapid development of gene drives systems is ahead of legal-ethical oversight, causing a “pacing problem” in this emerging field.^3^ Although this is more concerning for large-scale releases, current international and local governance is likely capable of proceeding with controlled field trials and releases in well-demonstrated geographically isolated areas. Anticipating release in larger, less-defined disease-endemic areas poses greater issues for governing bodies because of the capacity of drive organisms to expand beyond geopolitical borders, infringing on the consent of governments and communities alike.^4^ In an attempt to better equip the current international governance framework, new principles and strategies should be explored to enhance the oversight of future field testing and public trust of active genetic technologies. In support of international governance of mosquito gene drive technology, we examine the current regulatory bodies and organizations spread across the world that can have an impact on gene drive governance. These include those in the United States, Africa, India, Australia, Europe, and the World Health Organization (WHO). In many cases, legislation that directly addresses gene drive regulation is nonexistent but could be adapted from existing statutes in regulatory bodies and nongovernment organizations (NGOs) that handle standard genetically engineered organisms, as well as international agreements such as the Cartagena Protocol.^5^ Given the lack of precedent for wild release of gene drive mosquitoes, several strategies are proposed for the purpose of ensuring good governance, including the recommended use of Governance Coordinating Committees (GCCs); as well as the principles of transparency, accountability, participation, integrity, and capacity (TAPIC).^6^ The concept and reasoning of including additional coordinating bodies for gene drive governance has been expounded as the technological progression toward field testing necessitates it.^4^ The release of gene drive mosquitoes into wild populations will possibly be the piloting experiment of this nature, calling for focused and premeditated deliberation of international governance strategies.

## GENE DRIVE CONCEPT AND APPLICATIONS

Gene drive systems have the potential to bias the inheritance of specific genetic traits above that predicted by Mendelian genetics, which dictates that any given allele from a parent has a 50% chance of being passed on to an offspring. As a result, those inherited alleles showing gene drive will spread through the population over generations in either a self-sustaining or self-limiting mode depending on the construct design.^7^ The discovery of CRISPR-Cas9, a powerful genome editing tool, allows researchers to build gene drive systems to modify genomes at the population level through the propagation of genetic traits by non-Mendelian inheritance.^7^ CRISPR-Cas9–based gene drives work by cleaving the target version (often wild type) of a gene and promoting the cell repair mechanisms to copy an engineered version of the gene (containing the gene drive) into the damaged version. Autonomous drive elements convert heterozygotes into homozygotes, and the traits are passed on to all the progeny. The progeny in turn will convert to homozygotes for the drive, and this cycle could continue until the gene drive spreads throughout the entire population. Some drive methods use this technology in a spatial temporally limiting manner, such as daisy chain and underdominance drives, which are designed to be lost from a population following a period of fixation.^8^ All these systems could benefit from enhanced governing practices, particularly for self-sustaining “homing” drives, given their capabilities to rapidly modify populations. The efficacy of gene drive systems inherited in a non-Mendelian mode has been demonstrated in several diploid organisms with short generation times, including yeast, *Drosophila*, *Anopheles stephensi*, *Anopheles gambiae*, and mice.^9–13^

**Figure 1.**
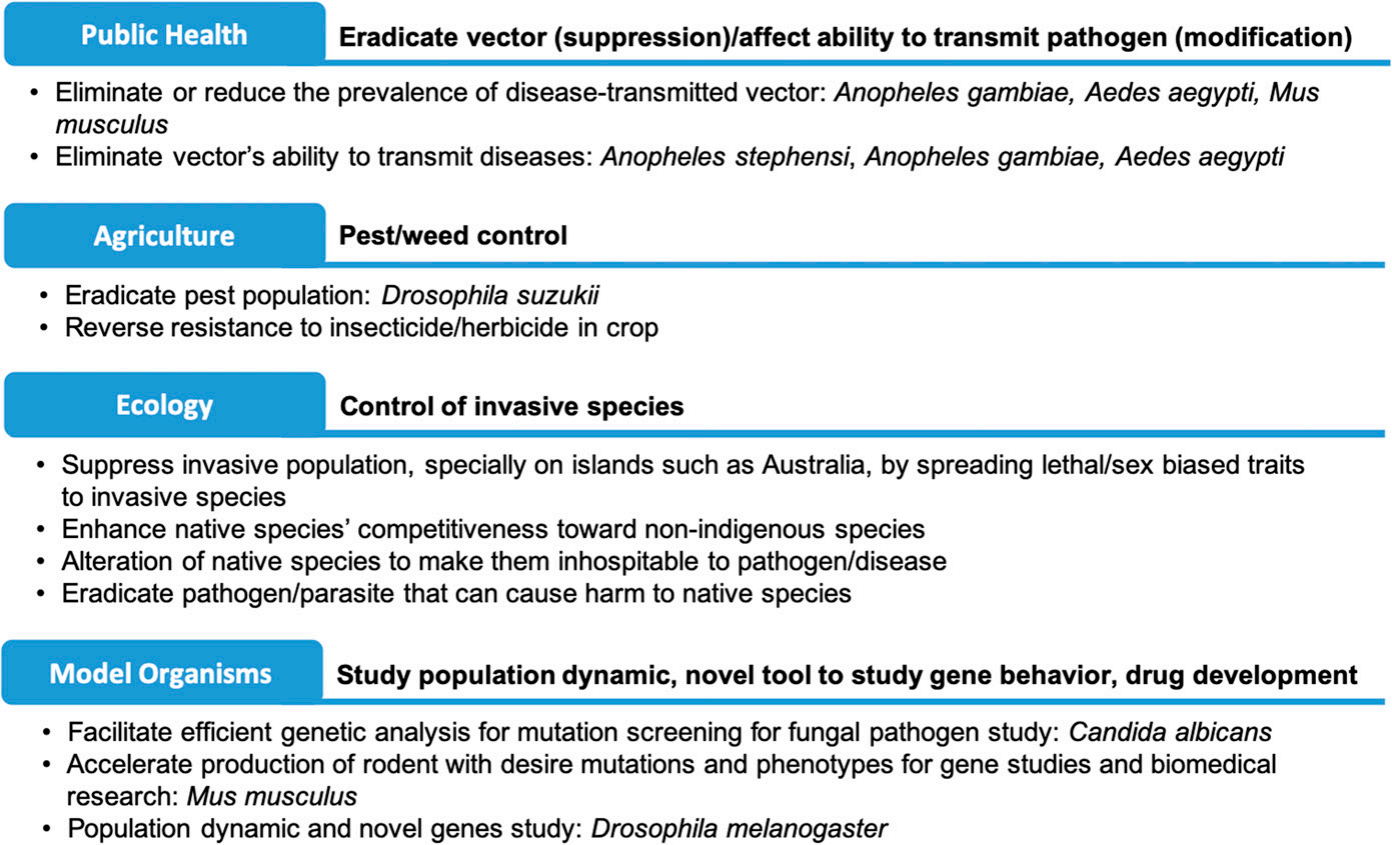
Potential and in-progress applications of CRISPR-based gene drive in genetically modified living organisms in different fields. This figure appears in color at www.ajtmh.org.

Recently, gene drives have been proposed as a potential tool to prevent the spread of disease pathogens through mosquitoes. Moreover, gene drive technologies can be adapted for pest control in agriculture and environmental conservation (eradication of invasive species) (Figure 1).^14,15^ However, research to develop gene drive tools is more prominent and advanced in the public health arena, with the development of gene drives designed to modify mosquito populations by introducing antimalaria genes that can block the malaria parasite transmission (population replacement) and gene drives that can eliminate mosquito populations (population suppression).^10,16,17^ Research in the Asian malaria vector *An. stephensi* has shown that gene drives in mosquitoes are capable of spreading antimalaria genes into laboratory cage populations within a few generations.^18^ This strategy is currently being adapted for the African malaria vector, *An. gambiae*.^19^ Moreover, researchers at Imperial College have demonstrated that gene drive can be used to eliminate a caged mosquito population.^20^ Furthermore, there also is an effort to introduce antiviral genes coupled to a gene drive system into the dengue vector, *Aedes aegypti*, to replace the wild mosquito population with pathogen-resistant mosquitoes.^21^

Gene drives in agriculture can be used for pest management to control insects by targeting essential genes in the pest genome. The current strategies focus on population suppression to eliminate the pest population.^22,23^ Another potential application of gene drive technology is environmental conservation by the eradication of invasive species, such as introduced rats on islands.^24–26^ Alternatively, the population modification concept can be used to use gene drives to spread beneficial traits or remove deleterious mutations in endangered species.^7^ With so many breakthroughs in gene drive success, it can be anticipated that the number of gene drive organisms will continue to increase, thereby prompting a need to analyze and identify relevant regulatory pathways and governance strategies that will facilitate field deployment.

## CURRENT STATUS IN GENE DRIVE GOVERNANCE

Governance is a hierarchical, authoritative framework that exists domestically and/or internationally. Within this framework, there are regulations that are collaboratively developed and mutually, democratically agreed upon or approved by a central authority. These regulations reflect a large number of collective ethical, socioeconomic, political, environmental, health and safety values, and concerns. Depending on the specific type of governance structure (on a federal level: democratic, monarchy, constitutional monarchy, etc.), the formal process by which these regulations are developed and ultimately implemented as law varies in methods of communication to affected constituencies and local governments (states, provinces, tribes, etc.).^27^

Currently, although the field of genetic modification is quickly growing, there is still no specific governance or process of rule-making on an international scale related to gene drives and the release of gene drive insects.^28^ This can be problematic and leaves innovative technologies vulnerable to being stymied by historical patterns of conflict among politics, scientific integrity, and corporate interest.^29–31^ Furthermore, exceptionalizing gene drive governance could foster conditions in which novel technologies will consistently require unique governance strategies that would be difficult to meet. Thus, incorporating a new technology into a preexisting governing framework is a more desirable and less protracted process. Potentially, satisfying the need for progress and governance would be modification or expansion of current governing bodies when encompassing a new technology like gene drive, to streamline regulatory efforts and account for the challenges posed by it.

Scientists remain on the cutting edge of this technology because of constant innovation and new discoveries in the field of genetic engineering. Therefore, it is critical that guiding principles are informed by a multilevel system in which federal governing bodies, leading global organizations in health and biomedical sciences and technology, geneticists, biomedical researchers, local governments, environmental and ecological scientists, and affected constituencies are all a functioning part of the international governance and informational process.^27,31,32^

### The United States.

Regulation in the United States of GMOs and subsequent gene drive organisms fall under the Coordinated Framework for the Regulation of Biotechnology, which includes the Food and Drug Administration (FDA), the U.S. Department of Agriculture (USDA), and the United States Environmental Protection Agency (US EPA).^33^ A lead agency is chosen in the case of a technology that is covered by multiple agency jurisdictions, as would be the case for gene drive arthropods. As a result of recombinant DNA being seen as a “new animal drug,” the anticipatory lead regulatory oversight for the release of gene drive mosquitoes might be considered through the FDA, in conjunction with the Animal and Plant Health Inspection Service of the USDA.^34^ The FDA released a guidance document in 2017 clarifying that jurisdiction for mosquito products that intend to function as pesticides for population control lies with the EPA, whereas those functioning to reduce or interrupt disease transmission remain as “drugs” under FDA jurisdiction.^35^ Depending on the intended use of other eventual “products” of gene drive research, their regulatory jurisdiction may change considerably. The most relevant and recent example of this regulation is the release of Oxitec’s genetically modified (GM) (non-gene drive) *Ae. aegypti.* Many of the guidelines given by the FDA for oversight of this project have the potential for being extrapolated into gene drive regulation, but these must align with public values and scientific rigor. Meghani and Kuzma criticized the FDA regulatory framework citing a narrow regulatory scope that excluded scrutiny of off-target effects to the ecosystem in favor of industry advancement and lack of acknowledgment for ethical concerns.^36^ Regulatory jurisdiction over Oxitec’s field trials has since changed from the FDA to the EPA because of the aforementioned federal guidelines. This change in oversight may ameliorate the concerns over ecological and ethical impacts experienced beforehand, as well as guide the U.S. gene drive regulation toward following the standards laid out by the National Academies of Sciences, Engineering, and Medicine (NASEM).^33^

The National Institutes of Health (NIH) has supported and prompted attention toward gene drive research and deployment. Multiple gene drive research programs are funded in part by NIH grants in an effort to stop disease transmission in endemic countries.^37^ Recognizing gene drive as a powerful tool, the Foundation for the NIH and the NIH requested NASEM to organize and report the current knowledge, applicability, and challenges of gene drive development and governance.^36^ Regarding gene drive governance, the committee emphasized that the government framework must promote community engagement efforts and open communication among stakeholders, communities, and the public.^38^ Moreover, there must first be an expansion of intellectual capacity to educate decision-makers and others responsible for authorizing the deployment of gene drive organisms. We support the expansion and inclusion of local institutions and scientists as an effective way of disseminating information to the community as well as connecting higher governing bodies to the release region. Such governing bodies should provide clear policies on how local concerns will impact regulation, risks, and research. All the governing principles provided by NASEM identify the progressive need for a future governance landscape, facilitating cooperation and developing the common ground necessary for safe and ethical deployment of gene drive organisms.

### Africa.

The African Union (AU) consists of 55 countries that focus on the unity and stability of Africa. One of the relevant agencies within the AU is the African Union Development Agency-New Partnership for Africa’s Development (AUDA-NEPAD), which provides opportunities for African countries to manage and develop agendas with international partners. To reduce malaria transmission in Africa, AUDA-NEPAD commissioned a high-level AU Panel on Emerging Technologies report.^39^ This report examines the malaria burden affecting Africa and searches for practical applications of gene drives at the country and regional levels. The gene drive techniques they suggest for malaria control are population suppression (sterile males) and population replacement (mosquitoes resistant to the malaria parasite). Along with the discussion of the strategies to control malaria, other elements of governance including risk analysis, management, policy, regulatory systems, and research and development are discussed in detail.

The involvement of AUDA-NEPAD continues with outreach opportunities where they provide meetings and workshops to discuss gene drives and their potential uses. With the assistance of the International Life Sciences Institute Research Foundation, regional workshops have been held in the Ghana (West Africa), Kenya (East Africa), Botswana (Southern Africa), and Gabon (Central Africa).^40^ In addition, a specialized program called the West Africa Integrated Vector Management Programme was set up to guide participants in better understanding gene drives and their use in future work with modified mosquitoes, in addition to developing guidelines for vector management including gene drives.^41^ Another group that held gene drive deliberations was the East African Community region, which anticipates that applications of new technologies such as gene drive can be beneficial as complementary tools alongside their current vector control programs. In their collaboration with different groups in Ouagadougou, Burkina Faso, AUDA-NEPAD convened with the West African Economic and Monetary Union where they discussed regulatory approaches of gene drive insects, transboundary issues, and the benefits of working jointly at the regional level.^42^ As of now, gene drive regulation has been placed under the biotechnology framework of the Commission of Economic Community of West African States.^43^

Other organizations in Africa involved with discussions on gene drives are the Pan-Africa Mosquito Control Association (PAMCA) and the African Academy of Sciences. Through PAMCA, Target Malaria (a program developing gene drives for controlling mosquito populations) provided a pre-meeting training course on gene drives targeted toward participants including researchers, policymakers, health professionals, and graduate students.^44^ The goal of this training course was to give these individuals the basic technical information about the uses of gene drives to facilitate discussions related to engaging with the public, regulatory concerns and risk analysis.^45^

### India.

India has been making significant contributions to the research and development of genetic engineering technologies. A regulatory framework was anticipated for emerging biotechnologies from an early start, with the Ministry of Environment, Forest, and Climate Change drafting and implementing the “Rules for the manufacture, use, import, export and storage of hazardous microorganisms, genetically engineered organisms or cells, ‘1989’ (or rules, 1989) under the Environmental Protection Act (EPA), 1986.”^46^ Although a signatory of the Cartagena protocol, India has not designated any genetic engineering technologies under the definition of a “modern biotechnology” as used in the Cartagena Protocol on Biosafety into a national regulatory authority, in lieu of legislative attempts. Therefore, this discrepancy allows complete regulatory authority to reside in the 1989 rules for a case-by-case approach to genetic engineering biotechnologies. The framework for the regulation of GMOs and release authorization comprises six competent committees with various responsibilities in the areas of advising, regulating, and monitoring. Subcommittees also can be initiated on a case-by-case basis for more specific purposes regarding review of biosafety data or monitoring of field trials. Approval for field testing of GMOs generally involves the various authoritative committees conducting a preliminary review of laboratory data and biosafety procedures (including risk assessment), proceeding to field site selection and facility inspection, and finishing with expert and stakeholder consultation before consideration for release by the Genetic Engineering Appraisal Committee. Special consideration for gene drives and other genome engineering technologies resulted in the Department of Biotechnology creating a dedicated Task Force on “Genome Engineering Technologies and their Applications,” which promotes research initiatives to harness the benefits of basic and applied use.^46^

The Takshashila Institution in India has provided a risk assessment report on testing and deployment of gene drives.^47^ It focuses on 1) the generation of GM mosquitoes, 2) deployment of GM mosquitoes through field trials, and 3) monitoring disease epidemiology, mosquito populations, and impacts in the food chain. At each stage, there is a focus on risk assessment and potential mitigations that can be initiated if any negative impact is observed. Regarding gene-editing governance, it promotes collaborative dialogue with an emphasis on set standards for safety to provide the basis for field releases. This discussion provides useful insights on the governance of gene drives not only for application of mosquitoes but also for other applications such as medical or agricultural purposes.^48^

### Australia.

The burden of mosquito-transmitted diseases on the Australian continent originates mostly from *Ae. aegypti*, which spreads dengue fever in Queensland, along with Ross River virus and Barmah Forest virus; together, these make up most of the mosquito-borne diseases.^49^ Because of the health burden of these viruses, Australia could benefit from gene drive development in arthropod vectors. Currently, there are two research groups working on gene drive technology in Australia, and these are regulated under the Gene Technology Regulations 2001 established under the Gene Technology Act of 2000 (GT Act).^50^ The Australian Department of Health enacted this legislation in response to growing concerns over GMOs with the purpose of safeguarding human and environmental health by regulating and managing risks related to genetic engineering. Administration of the GT Act is delegated to the Office of the GT Regulator, which is responsible for regulating, licensing, issuing procedural guidelines, monitoring risk assessment, and enforcing legislation. Through technical reviews, the GT Act is amended to currently oversee research on gene drive organisms, which it considers a higher risk endeavor requiring dealings not involved in intentional release licensing for contained research.^51^ This licensing ensures case-by-case evaluation of gene drive projects to provide optimal risk assessment and management to mitigate effects on human and environmental health. Alternative measures for lowering the risk level of the licensing have been proposed but not accepted in favor of maintaining more regulatory burden to commensurate risks.^49^

### Europe.

The governing body for GMOs on the European continent is the European Union (EU) as executed through the European Commission (EC). Through decentralized directives, the EC regulates various facets of GMO development, trade, and oversight.^52^ Although no specific laws governing gene drive organisms have been implemented yet, they are considered within the scope of GMO regulation. Gene drive organisms fit the laws that define GMOs on the basis of the introduction of sustainable synthetic heritable material through techniques such as micro-injection, as is used in gene drive arthropod vectors.^10,53,54^ The central directive regarding GMOs, including gene drive research, is the directive 2001/18/EC, also known as the “release directive.”^55^ It considers a case-by-case regulatory approach to the release of GMOs into the environment and requires an appropriate risk assessment evaluation before the release along with post-release monitoring activities. The main objective of this directive is to ensure that human health and environmental safety are not impacted by the release of a GMO. In addressing this objective, the European Food Safety Authority (EFSA) has direct involvement, as they have conducted a problem-formulating workshop for gene drive mosquitoes, among other guidance efforts for gene drive organisms.^56^ As the technology advances, the EFSA will continue to guide lawmaking, and industrial and research practices concerning gene drive applications. The remainder of the EU law on GMOs resides within directive 2009/41/EC on the contained use of GM microorganisms, which many member states have adapted to include GMOs in general.^55^ However, it is more challenging to classify gene drive organisms under this directive because it primarily relates to toxicological assessment and pathogenicity, neither of which relates to a gene drive insect with the exception of the potential toxicity of drive proteins, which should be studied for allergic responses. European Commission authorization only satisfies international access to release GMOs, as through directive 2001/18/EC, every member state can make independent decisions on the release of GMOs within their territory as long as they comply with EC directives.

On the state level, many countries have advisory boards on GMOs to frame regulations for research and release. The German Central Commission for Biological Safety (ZKBS) consists of an expert panel responsible for evaluating GMOs with regard to the potential risks posed to humans, animals, and the environment.^57^ The input from the ZKBS guides policy decisions on GMO development and regulation. Gene drive is a focus topic of the ZKBS, as they assess all research conducted on gene drive systems and advise on safety measures. From a proactive perspective, the ZKBS manages gene drive projects on a case-by-case basis, reporting directly to state authorities with safety assessments. An assignment of safety level (1 through 4) is given based on potential harm to humans and the environment, with higher levels bringing increased safety measures. An institute within the Dutch Ministry of Health, Wellness, and Sport is the National Institute for Public Health and the Environment (RIVM), which among many other responsibilities related to human health and environmental safety, has a role of ensuring the responsible development of synthetic biology.^58^ The RIVM also contributes in international issues of synthetic biology by participating in the Convention on Biodiversity, Organization for Economic Cooperation and Development, and the EU Commission on newly developing risks.

The United Kingdom also has its own organizations and laws governing the use and release of GMOs. A governance framework for environmental and human health involves the Health and Safety Executive (HSE), a nondepartmental public body that drafts, oversees, and enforces health and safety measures.^59^ With regard to GMO governance, the Scientific Advisory Committee on Genetic Modification provides guidance and risk assessment for GMO research and release, acting as the primary advisory body to the HSE on such topics.^60^ Together, they draft and oversee guiding principles for the release of GMOs. Key regulations such as “Genetically Modified Organisms (contained use) Regulations 2014” and “Genetically Modified Organisms (deliberate release) Regulations 2002” are the main routes of involvement for release and research.^56^ Regarding GMO releases, the Advisory Committee on Releases to the Environment (ACRE) provides the UK ministry with recommendations on consent approval for the release of GMO plants and animals.^61^ In addition, ACRE monitors release outcomes and risks as well as being a route for community engagement through subgroups. The deliberate release is allowed after an application process during which consent is given by the secretary of state in accordance with the EPA act of 1990 and Genetically Modified Organisms (deliberate) Release Regulations 2002.^62^ The EPA act of 1990, part VI intended to ensure that “all appropriate measures are taken to avoid damage to the environment that may arise from the escape or release from human control of GMOs.” These include limitations on the import, acquisition, keeping, release, and marketing of GMOs. These internal policies and procedures are not likely to be affected by the planned exit of the United Kingdom from the EU.

### Gene drive governance and international institutions.

International governance of gene drive technologies is important because regardless of how the technology is applied, multiple nations and organizations will have to communicate for contained trials and wild releases. International dealings with gene drive development will have to be sensitive to the socioeconomic and political difference between the nation providing and the nation receiving gene drive organisms to ensure stakeholder priorities and that associated interests maintain integrity.

The foremost international organization acting on the behalf of facilitating gene drive governance is the UN Environment Programme (UNEP). A subsidiary body of UNEP, the Convention on Biological Diversity, drafted, finalized, and ratified the Cartagena Protocol; the current mainstream international agreement on biosafety procedures for GMOs.^5^ It is meant to ensure the safe handling and use of GMOs intended to influence biological diversity, and that may pose risks to human health and transboundary movement.^5^ Member countries are required to have legislation, decision-making, and regulation of GMOs that adhere to the principles detailed in the protocol, thereby making this agreement an internationally unifying source of regulatory guidelines that can assimilate each country legislative structure to a core set of principles for responsible use of genetically engineered organisms.

The WHO has several groups focused on improving health practices and promoting health care that can influence international governance of strategies targeting vector-borne diseases. Hosted by the Special Program for Research and Training in Tropical Diseases (TDR), several meetings led to the publication of a guidance framework to establish best practices for research into genetically modified mosquitoes (GMMs) that include recommendations on biosafety, ethics, regulation, and efficacy, in addition to gene drive–specific recommendations.^63,64^ Moreover, the NASEM report specifically fitted the WHO guidelines for phased testing and risk evaluation to gene drive mosquitoes, providing more inclusive recommendations.^36^ The WHO limits its guidance mainly to immediate GMM features like efficacy and acceptability, whereas NASEM provides additional emphasis during phased testing to secure downstream resources necessary to scale up gene drive deployment.

The WHO Malaria Policy Advisory Committee (MPAC) performs essential scientific advisory and policy duties in an effort to control malaria and the vectors that propagate the disease. This expert committee has overseen various control strategies such as addressing a “global response plan” to *pfhrp2/3* gene deletions in *Plasmodium falciparum*.^65,66^ Assessment of control strategies is essential for advising current and future control models because they can have a direct influence over judging the target product profile for a gene drive mosquito and analyzing risk. In addition, the MPAC can influence the establishment of new committees for malaria control that may be essential for bringing gene drive technologies into international development. The Vector Control Advisory Group (VCAG) is the WHO advisory body on vector control methods, and their input is critical for moving gene drive mosquitoes into field trials. Currently, the VCAG supports gene drive technology and encourages further development, requiring extensive cage trial testing to gather substantial evidence to support field trial release.^67^

**Figure 2.**
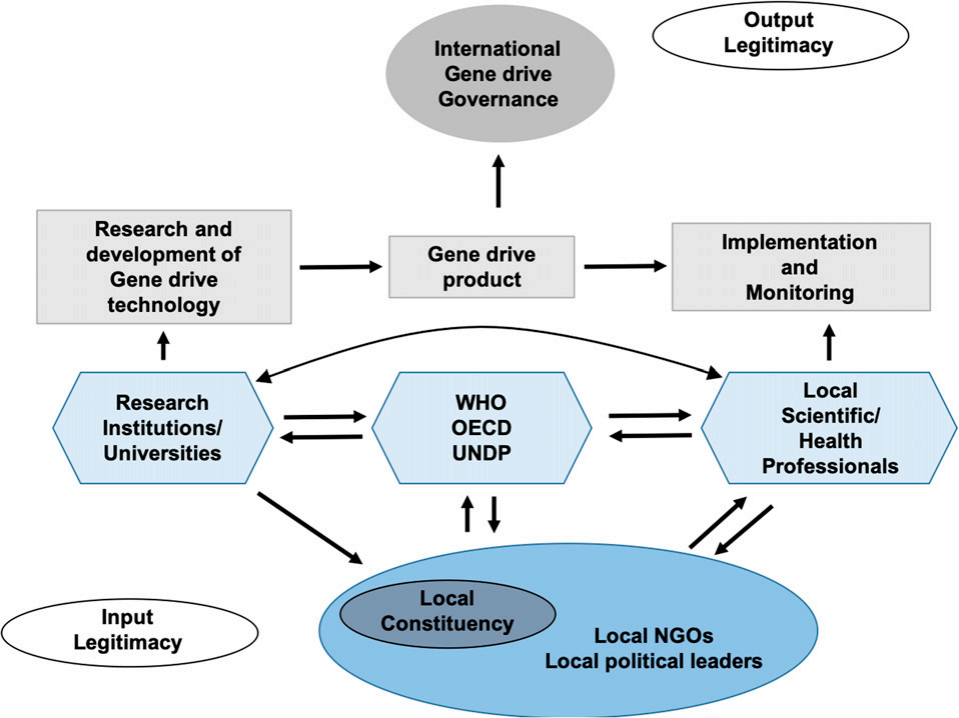
All participating governing bodies will oversee the release and monitoring of genetically modified populations. Efforts must invite the public into the governing process, through education and surveys of affected constituencies. Stakeholders must be mindful to avoid disproportionate focus on output legitimacy and performance: the genetic modified product itself and the actions of scientific and technological innovation, implementation, and monitoring separate from input and surveys of affected constituencies. Input legitimacy, defined as “collectively binding decisions (which) originate from the authentic expression of the preferences of the constituency in question,” is an absolute necessity.^27^ NGOs = nongovernmental organizations; OECD = the Organization for Economic Cooperation and Development; UNDP = UN Development Programme. This figure appears in color at www.ajtmh.org.

Given the history of genetic modification in relation to native and disenfranchised communities, input legitimacy, in the form of direct, mutual communication between governing authorities and local communities, along with public education, must be as significant a measure of gene drive success as output legitimacy and performance (implementation, monitoring, decreased levels of malaria incidences, etc.).^27^ An international framework that includes equal value of input and output legitimacy, and the governing bodies that form the connections between these two points, is illustrated in Figure 2. An international governing effort for gene drive use will likely involve WHO TDR participation, which provided a framework for vector control that is anticipated to be a guideline for participating countries’ vector control strategies.^68^ The Global Vector Control Response 2017–2030 (GVCR) was drafted with the aim of providing greater communication, coordination, and progression for the governance of new technologies.^69^ To reduce disease burden, the GVCR outlines the needs of improved vector response infrastructure, surveillance, basic research, and innovation. It recognizes that new technologies are key for combating disease and establishing a greater vector control capacity.

## COOPERATIVE GENE DRIVE GOVERNANCE

Constituting a governance framework for gene drive technologies will incorporate existing standard GMO regulations as well as adopting novel regulatory details to account for the special circumstances inherent in gene drive systems. For example, gene drives are capable of crossing geopolitical borders, which needs to be taken into consideration.^70^ In addition, an organism containing self-propagating genetic machinery may be capable of invasion into closely related (sibling) species.^71^ A successful international governance strategy must streamline communication between government and NGOs of the participating nations and develop multinational or/and bilateral agreements to mitigate the public relation challenges associated with these technologies. Furthermore, the groups of stakeholders (product developers and investors), public, and community require accessibility to each other to maintain accountability and respond to value-based concerns with scientific insight and innovation, which is a main objective of governance. This is achieved by numerous activities including risk assessment, regulation, and public engagement, among others. These responsibilities can be technically assigned to individual components within the governance framework, but having cohesive communication among participants is critical for review and to maintain integrity that ensures both public and scientific needs are met. The approach by which this is performed promotes public trust, gene drive legitimacy, and ensures gene drives are used on an “at-will” basis with philanthropic intent. Accomplishing this governance vision can be done through the use of GCCs to promote interfaces for the multiple bodies of governance and its stakeholders, allowing greater capacity and responsiveness.^71^ Rather than performing direct regulation, the GCC participates in monitoring, managing, and modulating gene drive technology through research and deployment.^71^ Monitoring goes beyond field release sites and into a more comprehensive evaluation of effective governance practices to identify problematic gaps. Management roles center around the GCC being an accessible resource to make efforts and issues publicly available, encouraging public engagement and allowing a comprehensive review of technological and governing strategies. Modulation is an important concept that requires significant collaboration, and a GCC would serve to align experts and stakeholders with the agreed-upon direction of gene drive technology as informed by the local populous, scientists, and regional authorities. Any changes need to be developed and executed in a manner that conforms to national regulatory processes, highlighting the need for inclusion of regulatory personnel from the disease-endemic country (DEC) in the GCC. The trajectory of a release project, and the technology itself, can rapidly advance and will require governing bodies to adapt and implement recommendations. Creating a GCC with the ability to promote timely reports, workshops, and best practices would require the cooperation of neighboring countries in authorizing its establishment drawing legal authority from a preestablished, recognized organization as a necessary condition upon authorization of the release of gene drive organisms. This condition could be integrated into bi/multilateral agreements between countries to induce a regional consent rather than a single country. Legitimacy for the GCC would be garnered from the recognized authority that it is established under, likely a regional authority on vector management. Using GCCs in the governance framework for gene drive can promote much needed public trust; knowing that proper collaboration and oversight are occurring can allow this powerful technology to be applied responsibly.

Along with the physical institutions of government, organization, and private establishments, an ideological framework also must exist as part of good governance of gene drive technologies. A thorough approach would involve incorporating a “TAPIC” framework: transparency, accountability, participation, integrity, and capacity.^6^ It is generally accepted that governance improves when the public has access to policy decisions and program directions. A questionable or nontransparent governance interface merely promotes skepticism and distrust, which can spread easily in the community. Workshops that make project details and discussions understandable, organized online resources, and explicit project checkpoints and reviews all help to maintain transparency.

Accountability promotes efforts on the stakeholders’ part to provide effective and safe products and release conditions. When the public sees respective organizations take responsibility for their science, it promotes a system of good faith that encourages public acceptance. It is a delicate balance to maintain proper lawful accountability, as an excessive or harsh backlash to possible gene drive problems could limit crucial progress and corrective innovation.

Participation from the public and community in gene drive governance decisions is essential, as their immediate insight can be the most accurate accounts of risks and benefits. In addition, this form of participation gives a proper understanding of how the community is perceiving and receiving gene drive technology, which in turn allows the stakeholders to accurately respond to public concern, making for more adaptive governance.^72^ A recent example of such action was AUDA-NEPAD’s implementation of workshops to identify protection goals and concerns among African stakeholders regarding the use of gene drives for reducing malaria incidence, which found that the potential impact on human health and biodiversity were the two major concerns.^39^

Integrity occurs in a subtler manner with regard to governance; it will involve providing clear expectations and goals of gene drive strategies made transparent by timely reporting. It also is pertinent to maintain integrity in the development of gene drive systems by properly deliberating conflicts of interest and patenting, which tend to occur in developing fields.^73^ Conflicts over intellectual property would only slow the progression of development and delay deployment, exacerbating the consequences of inaction.

The capacity of gene drive governance is related primarily to the available funding from both government and private interests. Currently, research in developing and testing gene drive elements within insect disease vectors receives funding from government agencies such as the NIH and Defense Advanced Research Projects Agency, in addition to private organizations such as the Bill & Melinda Gates Foundation and Open Philanthropy.^74,75^ The narrow target for each gene drive system in vector-borne disease control provides little incentive for private funding, although self-limiting gene drives are more attractive to private investment than are self-sustaining gene drives.^76^ Because of its technological infancy, much of the funding supports scientific advancement and understanding, and thus is mainly sourced from government and philanthropy. However, there needs to be more international funding and private involvement to expand to deployment. This is likely to occur as guidelines and governance develop to ensure responsible use and the investment return on gene drives, in both the form of financial return for stakeholders and reduction in disease burden. As funding expands with the scope of gene drive development, the governance network can maintain the capacity to deliver on the promises of gene drive systems.

Most of the discovery work researching mosquito gene drive strategies has been carried out in laboratories in the developed world with the intent to deploy the technology in malaria transmission regions in DECs.^17^ Under the guidance of the principles and components previously discussed, we describe here a theoretical governance strategy to explore the role of a GCC in an international governance framework for the release of gene drive mosquitoes in DECs. Four essential constituent bodies exist within this framework: 1) the source country, 2) the international organizations or governing bodies (WHO/UNEP), 3) the DEC governing authorities, and 4) the DEC local organizations. This composition provides direct links of the GCC to national and regional regulatory authorities, and the developers and users of the technologies. The recruitment of membership from these bodies into the GCC should be an integral part of the agreements on its establishment and should be representative of all major governing bodies including scientific, political, and management disciplines. The GCC will promote a close relation between the local authorities and the WHO to establish and assess monitoring drive assessment practices, release coordination, and public consent on an ongoing basis. Preemptive examination of the release geography and drive construct is essential, as a self-propagating drive will require greater input from neighboring countries than would a self-limiting drive with less expansive potential. In the form of timely reports and workshops, this information can be reported to the VCAG where review and recommendations can occur, with any changes ultimately being exercised through the GCC after local authorities and regulatory bodies’ agreement. Moreover, the GCC also can report to the local organizations who can provide management recommendations that can support current activities or drive new ideas for implementation. Input from the local population, organizations, and regional regulatory authorities is to be maintained in the recommendation and decision-making process executed by any international body, as to not be marginalized by international bodies. The types of information the GCC will report to the source country will have more emphasis on the performance of the drive construct to provide valuable data for population modelers and scientists relying on real-world data to improve the construct and make suggestions on their continued use and applicability. For example, if the release ratios are not sufficient, with the corresponding data, then the source institution and the VCAG with the corresponding data will be able to review release strategies and recommend changes to the local site of the DEC country through the GCC. Therefore, we perceive the opportunity for modulation of the drive project, a core component of the GCC, but modulation of governing practices also can occur through review of failing policies or oversight gaps. Modulation through critical, comprehensive, and intermittent review provided or communicated by the GCC highlights its capabilities of providing adaptive governance.

Contained field trial releases of gene drive mosquitoes, tested in outdoor large-scale but physically contained environments or in geographically isolated environments where the probability of dispersal should be near zero, will likely proceed in the near future without the involvement of the WHO or any proposed GCC.^33^ Authorizing GCC and VCAG oversight of a countries’ release project is a long-term goal that can be given incentive through endorsement of the gene drive product. Such a union could promote the technology to the public in a superior manner, signifying the trial is following and subject to rigorous international standards and guidance. Although not contingent for release, investigators and stakeholders should seek involvement from international organizations to move toward authorizing an enforceable GCC platform for governance that maintains project efficiency and upholds TAPIC principles.

## CONCLUSION

The successful use of gene drive technologies may replace failing practices and reduce the harmful impact that other vector control programs may have on the environment. The final purpose of gene drive technologies in public health is the release of modified organisms capable of reducing disease burden. The decision to use gene drive technologies must pursue broad consensus and cooperation from the nations that may be affected and must be based on rigorous evidence that demonstrates that the benefits outweigh the risks. However, this effort must not be limited to the ones delivering and receiving the technology, but should include adjacent nations to which modified organisms can spread and global organizations mediating the technology. In-depth knowledge of pertinent governing bodies across the globe will better allow the adoption of flexible gene drive governance strategies that address scientific, political, and ethical concerns. No two gene drive products are likely to be the same, so understanding the differences among constructs, environments, and target organisms must be considered for the governance needs, thus calling for an adaptive model of gene drive governance.
